# “Everybody breastfeeds if they have milk”: factors that shape exclusive breastfeeding practices in informal settlements of Mumbai, India

**DOI:** 10.1186/s13006-019-0204-2

**Published:** 2019-02-12

**Authors:** Sudha Ramani, Nikhat Shaikh, Sushmita Das, Shanti Pantvaidya, Armida Fernandez, Anuja Jayaraman

**Affiliations:** SNEHA (Society for Nutrition, Education and Health Action), Behind Bldg. No. 11, BMC Colony Shastri Nagar, Santa Cruz (W), Mumbai, 400 054 India

**Keywords:** Exclusive breastfeeding, Qualitative, Informal settlements

## Abstract

**Background:**

In India, though breastfeeding is universally practiced, exclusive breastfeeding (EBF) rates in urban informal settlements are low; and health programs face several challenges in promoting EBF. In this study, ensconced in one program area of a non-government organization, we focused on “positive deviant”- mothers who were able to practice EBF for six months and attempted to delineate factors that shaped their EBF practices. Typically, qualitative research from Lower and Middle Income countries on EBF has focused on understanding why women *do not* practice EBF; the converse perspective taken in this study has been less explored.

**Methods:**

We employed the positive deviance approach which contends that important programmatic learnings can be attained from persons who adopt positive behaviours. We conducted twenty-five diverse, purposively sampled case-studies of “positive deviant” mothers from two urban informal settlements in Mumbai; and analysed these using a framework approach. The results were summarised using a socioecological framework (consisting of individual, interpersonal, organizational and environment levels).

**Results:**

We found that mothers typically construed EBF as not giving breastmilk substitutes. Giving the infant minor supplements (water, honey) was not considered a violation of the EBF practice. The main themes that emerged as influencers of EBF included: at individual level, perceptions of having adequate milk; at interpersonal level, having role models who practiced EBF and having family support; at organizational level, advice from health workers (which was purported to play a secondary role); and at environmental level, financial constraints that limited access to supplements. One important finding was that women who practiced EBF could not always do it optimally; we encountered several instances of “*poor EBF*” practices, where mothers had breastfed infants inconsistently, allowing for long gaps between feeds, and had continued EBF even after six months.

**Conclusions:**

There is an urgent need for health programs to clarify the meaning of EBF and counsel against “*poor EBF*” practices. Messages received by women from immediate family on EBF were powerful and families play an important role in the actualization of optimal EBF practices. Hence, it is imperative to counsel entire families on EBF rather than women alone.

## Background

Exclusive breastfeeding (EBF) is defined as *giving no food to the infant except breastmilk except oral rehydration solution, vitamins, minerals or medicines for the first six months of life* [1]. Despite the recommendation of EBF for six months as a universal standard, and its proven links to improved child survival and reduced morbidity [2–5]; only 37–39% of infants in Low and Middle Income Countries (LMICs) are breastfeeding exclusively [6, 7].

In India, breastfeeding is a universal practice. India meets the globally recommended target for EBF [8] and recent national surveys estimate EBF rates in the country to be about 55% [9]. However, studies of urban informal settlements (slums) from different parts of India have estimated much lower rates, ranging from 8 to 37% [10–15]. Moreover, in the state of Maharashtra, where this study is located, urban EBF rates have been almost stagnant in the past decade, 49.9% in 2004 and 51.3% in 2014 [9, 16]. This study is based in two informal settlements of Mumbai (Maharashtra state, India), in the field areas of the Society for Nutrition, Education and Health Action (SNEHA), a Non-Government Organization (NGO) based in Mumbai that works in the field of Maternal and Child Health. A recent evaluation of one nutrition program at SNEHA had shown a positive impact on EBF rates at around 67% in intervention areas in the end line compared to 49% in the baseline [17]. However, program implementers at SNEHA have reported difficulties in enhancing EBF rates further- in spite of intensive counselling by programs frontline workers. This research was an attempt to derive some lessons for improving current program interventions on EBF.

In this study, we explored why some women were able to exclusively breastfeed, in an environment where other feeds like milk of other origin, formula, water, and traditional preparations were commonly given to infants before six months of age. This approach was rooted in the ideas of “positive deviance” evaluation [18] which contends that important program lessons can be learnt from people who adopt positive behaviours. In our study, we defined positive deviants as mothers who were able to practice EBF for six months.

### Current literature on EBF and gaps

A vast expanse of literature exists on factors that affect EBF practices from diverse contexts. Quantitative studies have shown that EBF rates were influenced by mother’s education, age, and employment; infant’s age, sex; access to healthcare; neighbourhood of residence; and exposure to mass media or counselling [19–23]. The direction and magnitudes of these associations were, however, not universal, and these point towards a strong influence of context-specific mechanisms on EBF practices. One of the most cited barriers to EBF across different types of studies and contexts- was the perceived inadequacy of breastmilk for the child [12, 24–26]. While studies have highlighted lack of knowledge on EBF practices as a barrier [11, 27], there was some evidence that mothers did not practice EBF despite having knowledge [28–30]. In addition, the recent growing body of qualitative studies has been particularly useful in delineating nuances of factors affecting EBF practices. In Zambia, in the context of high death-rates due to HIV, the fear of dying and leaving behind a child dependent only on breastmilk has been reported as a strong barrier to the practice of EBF [31]. In urban Kenya, the experience of hunger led mothers to perceive their milk as insufficient; and food insecurity appeared to be seminal in deterring EBF [28]. In Pakistan, mothers adhered to traditional practices of giving prelacteal feeds despite medical advice given against these [32]. In Lebanon, women have expressed deep-rooted cultural concerns about breastmilk having the potential for being “bad milk” that might be nutritionally inadequate or even harmful to the baby [33]. Clearly, the practice of EBF and factors influencing it are rooted in the sociocultural, geographical and economic milieu in which mothers live.

However, so far, qualitative research in this field has typically focussed on understanding why women *do not* practice EBF. The converse perspective, what enables women who EBF to do so, has been less explored in literature from LMICs. We found only one study that focussed explicitly on a woman who practiced EBF [34]. We felt that focussing on women who practiced EBF in our setting, and elucidating factors that enabled them to do so would yield rich insights into the practice of EBF, and thus, enable programs to design more contextualized interventions on breastfeeding.

## Methods

### Research methods

An exploratory qualitative research design was used, wherein the perspectives of mothers who exclusively breastfed their infants was sought through in-depth interviews.

#### Study setting

This study was conducted among mothers residing in two selected areas within urban informal settlements in Mankhurd and Govandi, Mumbai city (see Table 1). In these areas, the programs implemented by SNEHA had been running for six months at the time of the study.

**Table 1:** Demographic and health characteristics of the population from the urban informal settlements chosen for the study

Characteristics	Mankhurd	Govandi
Household Characteristics		
Total number of households	603	692
House ownership – rental	334 (55%)	336 (49%)
Average household size	6	5
Average no of children in a household	3	2
Religion		
Muslim	500 (83%)	442 (64%)
Hindu	100 (17%)	244 (35%)
Others	3 (< 1%)	6 (< 1%)
Education		
No formal schooling	225 (37%)	192 (28%)
Child statistics		
No of children under 2 years identified	411	442
Institutional delivery	365 (89%)	412 (93%)
Low birthweight^a^	57 (21%), *n* = 275	86 (23%), *n* = 369
Nutritional level^b^	*n* = 369	*n* = 375
Wasted	56 (15%)	81 (22%)
Stunted	149 (40%)	167 (45%)

^a^- based on available data for birthweight

^b^ - based on available data for anthropometric measurements

*Source: Baseline report of the study area, 2016*

#### Selection of participants

To identify mothers who had exclusively breastfed their children, we used data from a baseline survey done in March 2016. The initial plan for participant selection was to use the baseline survey to locate mothers who practiced EBF, and select a purposive, maximum diversity sample from this group. However, in the field, we found that several mothers, who had been identified in the survey as practising EBF, had actually given some supplements. This overestimation could be attributed, to a large extent, to the use of a single 24-h dietary recall interview technique used in the baseline survey to estimate EBF rates. Other studies have also reported similar overestimations [35, 36]. Hence, we modified our participant selection strategy; we used a list of mothers who practiced EBF as per the survey, and further verified with them if they had given any supplements to the infant before conducting the interviews. We purposively selected participants from different age-groups, parity, education and household structure, since we felt that this heterogeneity would help illuminate different dimensions of the EBF phenomenon (see Table 2). For this study, we conducted 25 interviews; after the first 15 interviews, we did not come across major new themes. Since the program area was new, participant mothers’ exposure to advice from community health workers of our program was limited (some had been visited 1 or 2 times by the program workers).

**Table 2:** Sociodemographic characteristics of the study participants from the selected urban informal settlements, Mumbai (*n* = 25)

Sociodemographic characteristics	*n*
Age (years)	
18–25	12
26–30	8
> 30	5
Religion	
Hindu	5
Muslim	20
Education	
Less than 6 years of schooling	15
6–12 years of schooling	8
Graduates	2
Employment status	
Not currently employed	25
Migration status	
Migrants	18
Not migrants	7
Household structure	
Nuclear family	11
Joint family	14
Parity	
Primiparous	6
Multiparous	19
Themes covered in In Depth Interviews with mothers who exclusively breastfed their infants • How mothers understood EBF • What made the mothers take the decision to EBF- as per their definition of EBF • Cultural values and beliefs regarding infant feeding practices • Major sources of advice on EBF given to mothers (from family, media and the health system) • Perceived enablers of EBF for 6 months; how and why could these mothers practice it when others could not. • Perceived barriers to EBF and how these mothers coped with these barriers	

#### Data collection

**Fig. 1:**
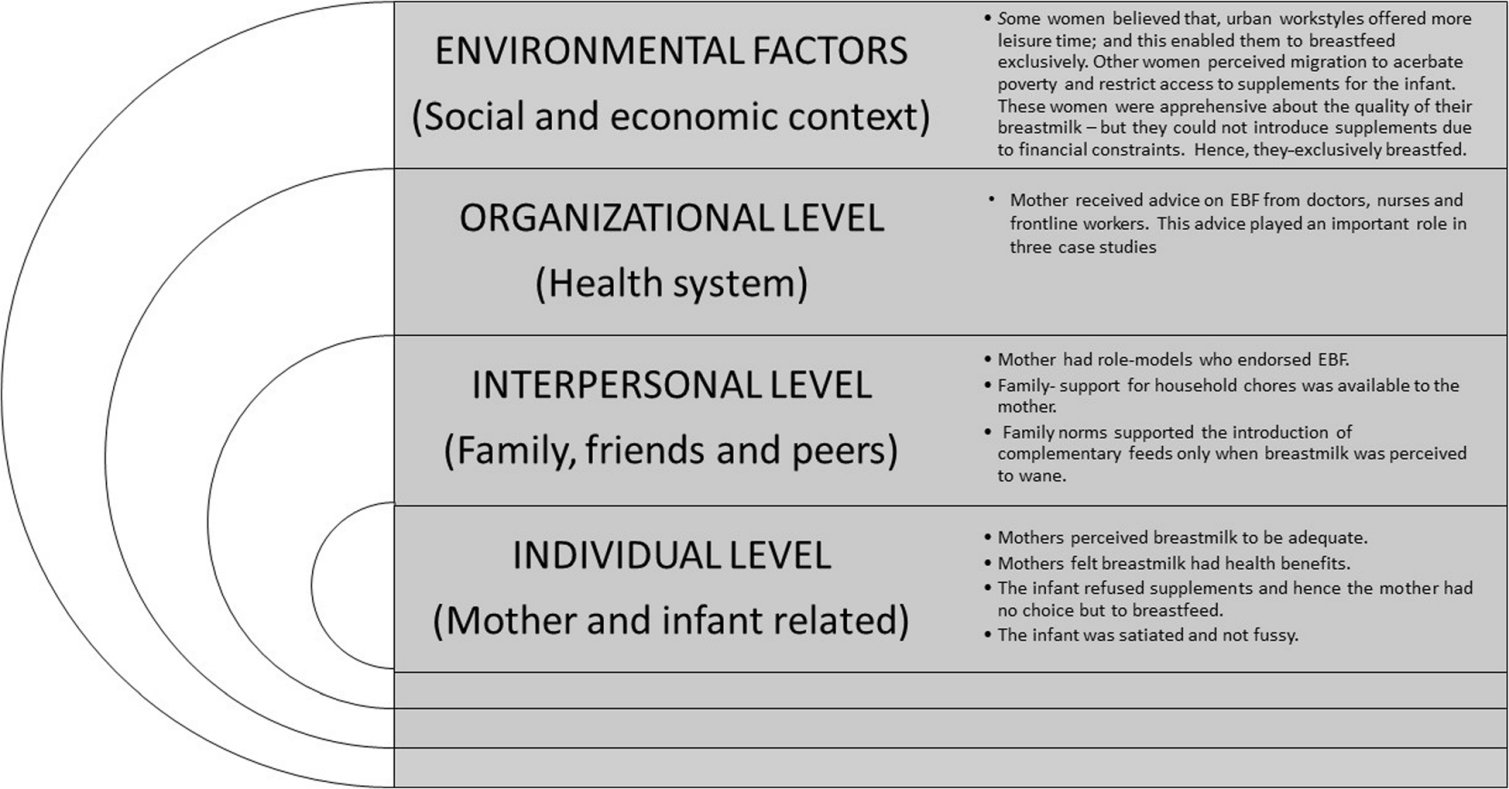
Why mothers did not give major breastmilk supplements

Authors 1 and 2 conducted the interviews with mothers between January–May 2017 (One research intern also accompanied them for some interviews, but she did not conduct any interviews independently). Interviews were conducted in Hindi and Marathi within the houses of the respondents. The average duration of the interviews was 45 min. Participant mothers were asked about their understanding of EBF; sources of information and advice on EBF; and about barriers and enablers to the practice of EBF (see Table 2 for a summary of themes). All interviews were voice-recorded, translated and transcribed verbatim into English, and pseudonymized. We analysed the data through “framework” analyses techniques [37], wherein the analytical emphasis was on drawing interpretations through data reduction (selecting and sorting data systematically) followed by data display (organizing the sorted data into a case by theme matrix). For data reduction, the two researchers who had collected the data sifted through the first eight transcripts, affixed preliminary codes, and developed a coding index. Initial data analyses suggested that the themes that emerged in our study could be organized into a modified socioecological framework (refer Fig. 1). This framework contends that behaviours such as the practice of EBF were influenced by the interaction of factors at various levels: individual level (mother and child); interpersonal level (family, peers and friends); organizational level (systemic interventions on health and nutrition); and environmental level (social and economic factors) After the initial framework was discussed and agreed upon by all study investigators, two researchers independently coded the data using NVivo (version 10), and discussed dissimilarities, to ensure consistency in the application of the coding process.

## Results

### Defining EBF for the study

There was no literal translation available for the term “exclusive breastfeeding” in the local language; participant mothers were asked “*You did not give anything to your infant besides your breastmilk for 6 months. Could you please tell us why?”.* Initial analyses revealed that for many women in our context, EBF primarily implied not using breastmilk substitutes (milk of other origin, commercial formula or semi solids). Many mothers, who had occasionally given the infant water, prelacteal feeds like honey or traditional medicines- claimed to practice EBF; since these were not considered as “substitutes” for breastmilk.

Despite our best efforts to screen out mothers who had given water, honey or traditional medicines to the infant, we found during our detailed conversation with them that some of these had been given occasionally- though in minimal quantities. Two mothers had given honey and sugar-water as prelacteal feeds; some mothers had given water only in summer; and traditional medicines like *ghutti* (herbal tonic) were used if the infant had stomach-ache. Some mothers could not recall with certainty. Due to issues of recall, we only analysed why these mothers did not resort to giving major breastmilk supplements during the first six months.^1^

### Why did mothers not give major supplements to the infant for 6 months?

We have used a socioecological framework to summarise the reasons mothers put forth for practising exclusive breastfeeding (Fig. 1). The different factors are detailed below:

### Individual level

#### Perceived adequacy of breastmilk by mother

In twenty cases, mothers perceived themselves as having adequate breastmilk to satisfy the infants’ needs and hence did not introduce supplements. There was a strong belief that breastmilk was God’s gift and only women who did not have breastmilk must resort to supplements. Mothers perceived their diet to be linked to the quality and quantity of breastmilk produced by them. Women who exclusively breastfed tried to take additional care of their diet; and sometimes took herbal supplements to make their milk better:

*“For milk production, I eat a lot of pulses. They say- if you drink porridge made of pulses, there is increased milk flow; so, I drink porridge made of different pulses. Then I eat fruits, a cashew-almond mixture… so that the child gets good milk. So, I have a good flow of milk.”* (Age 20 years, two children).

The perception of having adequate breastmilk was strongly linked to the time of introduction of complementary foods:

*“I will feed depending on the availability of milk. If milk is there for 6 months, then I will give for 6 months. If milk is not produced, then I will give outside milk … (milk from other sources).”* (Age 22 years, one child).

As long as breastmilk was perceived to be available, mothers did not feel the need for introducing complementary feeds. This had an unintended negative consequence, since several mothers in our sample did not stop practising EBF even after six months.

#### Perceived benefits of breastmilk

Most mothers were aware of the benefits of breastmilk, and pointed out that breastmilk made children strong and imparted immunity:

*“Because of breastmilk, there is strength in the body. Otherwise, when kids are born*. *.*. *they fall, their hands and legs break, outside milk doesn’t have that much strength. The strength (that) is there in the mother’s milk, that is obviously not there in outside milk.”* (Age 35 years, four children).

Some women felt that supplements were difficult for the infants to digest. However, while mothers pointed out the benefits of breastmilk, they could not tell us the benefits of EBF specifically.

#### Babies refused supplements

Eight mothers reported that they had tried to introduce supplements (either cow’s milk or popular commercial brands of supplements); but the infant had refused to comply and hence they had no choice but to breastfeed exclusively. Supplements were generally tried by these mothers during times when they had felt that their breastmilk was inadequate or when the mother had other chores to attend to and did not have time to breastfeed.

#### Infant was satiated

Most mothers took cues from the behaviour of the infant to introduce supplements. Mothers reported that the infant being happy, sleeping well, and not demanding food, were signs that it was getting adequate nourishment.

### Interpersonal factors

#### Role models who breastfed exclusively

In the study, almost all women breastfed because they had seen it being practiced in their families; mothers, sisters, mothers-in-laws and sisters-in-laws were role models for breastfeeding. The norm was to breastfeed exclusively as long as milk was perceived as adequate. Some participants reported that their families encouraged them to continue breastfeeding-even during challenging circumstances such as ill-health. Table 3 delineates the case of a woman who coped with her anxiety of having insufficient milk and practiced EBF due to reassurance from immediate family.

**Table 3:** Case study of a woman who exclusively breastfed because it was a family endorsed practice; she had family support; as well as the financial resources to cope with the anxiety of milk inadequacy

The woman lived in a nuclear family, close to her mother’s house. She had three children. She had been told by her mother to give only breastmilk to the child. She had also seen other family members breastfeed their children.	
*“Be it my brother’s wife or my brother-in-law’s wife, they have given only breastmilk to their babies. In our family, it’s like this only”*	
The woman felt that it was necessary to have good quality breastmilk in order to feed the infant. She took care of her diet specifically by eating lentils, milk powder and green vegetables.	
She was anxious about her milk waning as the infant grew. She bought a tin of formula, but the infant did not eat it. So, she desperately resorted to eating supplements (herbal medicines) to increase her milk production:	
“*I didn’t even have milk but what to do, he used to cry, but still somehow, I breastfed him for 6 months. I had medicines, so that milk production would happen.”*	
The woman felt great pride in her ability to breastfeed her child exclusively. Her mother had told her that in this generation, this was rare. She wanted to breastfeed her child for as long as she could:	
*“When I went to the hospital, I saw people coming with their two months old with a bottle. I used to look at them and think that I should try and feed my child my milk as long as I can.”*	
The woman claimed that it was her mother’s support in doing household chores that enabled her to practice EBF. Her mother helped by washing clothes, cooking food and taking the other children to school. So, the woman herself could concentrate on the infant’s needs and feed him (and herself) well.	

#### Family support for EBF

Eighteen mothers reported some form of family support which they perceived as essential to their ability to practice EBF:

*“Yes, I think without their (family) support, it would have not been possible to exclusively breastfeed my children. Without them, I would not be able to do this*. *.*. *should I cook food or look after the children or do household chores, or drop (the other) children to school? You get caught up with several such things*. *.*.” (Age 23 years, having three children).

Participant mothers reported receiving support from their family, in terms of post delivery care, provision of cooked food, care of other children, and other household chores.

### Organizational factors

#### Advice from professionals

Twenty women reported being exposed to some form of medical advice on EBF from medical doctors, nurses or community health workers. Advice from doctors and nurses was given at the hospital during delivery, and women reported feeding colostrum and avoiding external prelacteal feeds in accordance to this advice. Women revealed that community health workers who did home visits also advised them on EBF. Some mothers also mentioned a government phone application which sent them reminders on breastfeeding and immunization.

However, despite being exposed to advice on EBF from various sources, we found that, usually, this advice was not central to women’s decisions on the practice of EBF. Exclusive breastfeeding decisions appeared to be based primarily on factors such as family support and endorsement and perceived availability of milk, rather than on professional advice. Only in three cases, we found that women had gone against their family norms (of introducing other feeds) and had followed medical advice on EBF. In one case, the mother was a graduate and she acknowledged that medical advice had value. In the other two cases, the mothers had previously experienced deaths of their infants; and this experience made them more amenable to medical advice (see Table 4 for an illustrative case).

**Table 4:** How an adverse event in a woman’s life induced her to defy family norms, listen to medical advice and practice EBF

This case was of a 31-year-old woman with three children. She had lost two of her children in infancy. She attributed this loss to listening to poor advice from family on breastfeeding.	
She had given birth to her first child in a rural area and post-delivery, she had to do many household chores. She felt very tired at night. Seeing this, her sister-in-law had advised her to start bottle feeding the infant when it had been only 15 days of age. Following the bottle feeds, the baby had succumbed to frequent episodes of diarrhea and had died. She had thereafter migrated to Mumbai and had been blessed with a second child. The second child had been fed minced almonds on the advice of a relative who had felt that the child was very thin. This child had also died. The second loss made the woman determined not to follow advice given by her family. In her own words,	
“*From then, I made a decision, not to give my infant anything. I go to the doctor, whatever he says, I follow that. My husband shouts a lot, my family says something or the other*. *.*. *do this and do that. But I don’t listen to them, only to the doctor. I had made up my mind”*	
She believes now she had made the right decision in listening only to the doctor’s advice on EBF. Now she has three children. The mother also felt that migration to Mumbai helped her since it eased her workload. Also, staying away from her family reduced interference from her relatives, which enabled her to follow medical advice more easily.	

### Environmental factors

#### Social and economic context

Women in our sample referred to urban migration and poverty as factors that affected their decisions to exclusively breastfeed. We encountered two pathways through which these contextual factors affected EBF. One, some mothers perceived that urban transfers worsened poverty, due to lack of job security of spouse, lack of financial support from immediate family, and inability to withstand sudden crisis. Under these circumstances, mothers felt that their diet was considerably restricted and that their breastmilk would be insufficient for the child. However, these women also felt that they could not always afford to buy supplements and hence they breastfed exclusively. In such cases, we often came across *“poor EBF*” practices, where the mother did not feed the infant consistently; or on demand; and left long gaps between feeds. (Tables 5 and 6 illustrate cases of poor EBF practices).

**Table 5:** Case of a mother, recent migrant who struggled with issues of basic survival and poverty, and believed she had no option but to exclusively breastfeed

This case was of a 32-year-old woman who stayed in a rented house with her husband and five children. Her husband drove an auto (a three-wheeler cab) for a living. In the previous year, a fire had destroyed their house- and all their possessions.	
*“You must have heard about the fire, for one month my children were here and there, they didn’t get proper food or milk, because of that she (the baby) fell ill. After that, she didn’t get well at all, she had lost so much weight. I could not give her a massage. We didn’t have a house. There was problem in food, water, bathing”.*	
This mother believed that she had no option but to practice EBF. She could not afford to buy supplements. She did not believe that her breastmilk was adequate for her child since she perceived her own diet to be inadequate. She expressed worries about her child falling sick often and wondered if this was due to inadequacies in her breastmilk. In her words:	
*“From the time she was 3–4 months old, she keeps getting cold and cough. I think if she eats rice and lentils, she will not fall sick. Maybe my milk does her harm”*	
In this case, it was clear that the woman did not believe in the benefits of EBF; but she practiced it since she perceived herself as having no choice.

**Table 6:** Case of a woman who was abused; had a malnourished infant and reported practising EBF in an inconsistent manner

The mother was a 20-year-old woman, who had undergone 5 abortions, two after the first child and three after the second. She had married against the wishes of her family and was alienated from them. She was separated from her husband due to issues concerning domestic abuse. She believed that both, she and her kids, were weak because of the repeated abortions she had to undergo.	
The mother felt that she had not been able to take care of her children due to the tensed atmosphere at home, and frequent quarrels with her husband. When frustrated, she often left home abruptly, leaving the baby unattended. During such times, the baby had been given neither breastmilk nor substitutes. In her words:	
*“When his father used to fight with me, I would take the anger out on the baby. I didn’t give him milk, I didn’t give him a massage. I feel I should not have done this, what fault of the baby is it?”*	
The woman reported exclusively breastfeeding the infant, that is, giving the infant nothing except her milk but she also acknowledged that she did not breastfeed consistently.	

In contrast, we also came across some mothers who felt that migration to the urban region had actually improved their economic conditions. They perceived urban workload and lifestyles to be easier than rural ones. (Table 4 illustrates a case where, in addition to other factors, the mother felt that her migration to Mumbai was conducive to the practice of EBF, since her workload was easier, enabling her to concentrate on child-rearing.)

The infant was exclusively breastfed till about she was a year old. The mother also admits that she was often not able breastfeed frequently, and was pre-occupied with other household issues.

## Discussion

This study contributes to extant literature on EBF in two ways. Firstly, previous research on EBF has not given explicit and focussed attention to women who practiced it. We felt that the perspectives of women who practiced EBF and the range of factors that enabled them to do so called for detailed, nuanced exploration. This has been attempted through the 25 rich and diverse case-studies of women in this paper. Secondly, studies on EBF have typically been structured around the technocratic definition of the concept. However, recent literature has pointed out that mothers do not really understand the term “EBF” clearly [26, 38]. In this study, we have attempted to derive a community-contextualized definition of EBF, and hence, examined how mothers practiced it. In our study, women mainly understood EBF as not giving breastmilk substitutes such as milk of other origin or formula or complementary food- to the infant before six months. Giving minor supplements such as occasional sips of water was not considered a violation of EBF practices.

Some of our findings on factors affecting EBF reverberate with those found in other qualitative studies. The need for perceived adequacy of breastmilk has been reported consistently in studies across diverse cultural settings and contexts [12, 24–26, 39]. In our sample, we also found an underlying anxiety about the adequacy of milk for the infant. Most women in our sample coped with this anxiety by strengthening their diets as best as they could.

The importance of family support for breastfeeding and the existence of “role-models” who practiced EBF have also been seen as important enablers [32, 40, 41]. We also found this to be the case in our study. In our study, the key source of knowledge for women on breastfeeding was family. The practice of not giving other milk or formula, as long as breastmilk was perceived as adequate, was culturally sanctioned rather than advocated for the biomedical benefits that EBF had. Mothers were generally aware of the benefits of breastmilk, though not specifically about EBF.

Literature from other contexts has shown that access to healthcare in the antenatal and postnatal periods, as well as counselling and exposure to mass media promotes EBF [11, 21, 42, 43]. A study exploring factors that led to improvement in EBF rates among women enrolled in a large-scale child nutrition program found that EBF rates were higher among those who received counselling services from community health workers and attended group sessions offered by the program implementers [44].

Many women in our study reported receiving messages on EBF from health professionals, particularly doctors and nurses during the time of childbirth. However, they did not perceive messages from these professionals as strong influencers of their decisions on EBF. Only when confidence in traditional ideas of feeding the child was broken due to adverse events, was importance given to medical advice on EBF (we encountered two such cases). In general, the study findings implied that messages from professionals play a secondary role in comparison to the messages on breastfeeding mothers receive from immediate family and peers. Also, our findings indicated that support from immediate family plays an important role in the actualization of optimal EBF practices. All this clearly underscores the need to counsel entire families on EBF practices, rather than mothers alone.

We used a broad socioecological framework for analysing factors affecting EBF (similar to Hector et al. 2005 [45] and adapted it as themes emerged from the case studies. Such ecological frameworks have been used in previous breastfeeding literature from LMICs [28, 30]. This study reiterates the applicability of socioecological frameworks in studying EBF practices. In addition to distinct factors in the framework, we felt that it was important to acknowledge the complex interactions between these factors that led to EBF. Some of these interactions have been captured in the four illustrative case studies. These case studies explicate how, under different circumstances and with different mothers, the factors in the ecological framework interact, negate or complement one another to influence EBF practices.

We paid attention to two broad contextual factors, poverty and migration, since these emerged frequently during discussions with study participants. The overarching role of poverty and subsequent food insecurity in influencing EBF has been underscored in literature [28, 46, 47]. In our context, poverty and migration combined to influence EBF through two broad pathways. Some women believed that urban migration had improved the family’s economic prospects as urban workstyles offered more leisure time than rural, and this enabled them to spend more time with the infant and practice EBF. Other women perceived migration to exacerbate poverty and further restrict access to food and supplements for the infant. These women practiced EBF despite being apprehensive about the quality of their breastmilk, because they believed themselves to have no choice. Akin to this mechanism is the one described by Lesorogol et al. 2017 [47] as “*last resort EBF*” in Haiti, wherein poor economic conditions forced women to EBF.

A combination of poverty, lack of access to financial resources, being young, having multiple children, and sometimes dealing with violence puts extreme pressure on mothers who practiced EBF as a “*last resort”.* Exclusive breastfeeding in such cases, was done mainly because mothers perceived themselves as having no choice without an explicit understanding of EBF and belief in its value. These mothers often did not do justice to the practice of EBF as presupposed in international prescriptions of the concept. Mothers reported and we observed instances wherein children were left unfed for long hours (Table 5 and 6). Such EBF practices can have adverse effects on the health of infants. Given this, we feel that in contexts similar to ours, it is important to not just advocate EBF, but focus strongly on “*good EBF”* practices. Women need support not just to practice EBF, but to practice EBF *well,* in a manner that is optimal to the infants’ health.

We discuss below two limitations of our study. First, it was difficult for women to recall the use of minor supplements with certainty. We feel that a different approach such as longitudinally following up with women to see if they practiced EBF, might elicit better evidence on minor supplements. The methodology we used worked best when we defined EBF from the women’s point of view, as not giving major breastmilk substitutes, rather than adhere to a strict technocratic definition of EBF.

Second, in the cultural set up of the particular urban informal settlements we drew our sample from, women were generally not employed outside the house. Women, during certain seasons, took up part-time work like embroidery on clothes/stitching to earn some income, but rarely left the house. Women in these settings were mostly dependent on their spouses for finances. Hence, we could not explore links between mother’s employment and EBF, an aspect that has been seen as important in other contexts [23, 26, 28].

## Conclusion

Table 7 presents key learnings for health programs from our study. In summary, we perceive a strong need for messaging on EBF in our programs and beyond so as to explicate the meaning of EBF in communities. Messages must concentrate on the promotion of “good” EBF practices, like feeding on demand and with consistency; and also address issues of late weaning. Programs need to focus on counselling families rather than only the mother on EBF.

**Table 7:** Key messages for health programs

Program lessons	Description
The need to explain EBF clearly	Our findings indicate that the conceptualization of EBF in the community was different from its technocratic definition; women did not consider giving minor supplements to the infant as a violation of EBF. Health awareness messages need to clarify the meaning of EBF in communities.
Challenges in estimation of EBF rates	We found that women who had reported that they practiced EBF in the program baseline data, on detailed discussion, had not actually breastfed exclusively. In addition to clarifying the meaning of EBF, there is a need to modify the single 24-h recall technique of questioning women about EBF currently used in our programs.
The need to address suboptimal EBF practices and late weaning practices	Despite practising EBF, some women in our sample did not breastfeed on demand and did not know how often to breastfeed the child. We encountered cases where the mother had neither breastfed the child consistently nor given breastmilk substitutes. It is important for health messages to convey how EBF can be “done well.”, and to promote the introduction of complementary foods beyond 6 months.
EBF as a family decision	This study shows that immediate family is an important influencer of EBF. All awareness messages on EBF must be directed at families rather than mothers alone so that the mother gets optimal support to practice EBF well.

## Abbreviations

EBF: Exclusive breastfeeding
LMIC: Lower and Middle Income Countries
NGO: Non-Governmental Organization
SNEHA: Society for Nutrition, Education and Health Action

## Footnotes

1 Eleven mothers among our participants confidently denied giving their infant any minor supplements. The reasons given by them were: Other family role models did not use any such supplements; The infant was born during winter/rainy season and did not need water; The infant did not get any stomach ache or colic- and hence did not need supplements; Honey and other traditional medicines were not affordable. Women also reported receiving medical advice against minor supplements from doctors and nurses in the hospital when they delivered. This advice appeared to play an important role in minimising pre-lacteal feeds when women were in the hospital after delivery; but was not taken very seriously thereafter. Due to difficulty in recall, we could not analyse these results in detail.
